# Ecological Drivers of Species Distributions and Niche Overlap for Three Subterranean Termite Species in the Southern Appalachian Mountains, USA

**DOI:** 10.3390/insects10010033

**Published:** 2019-01-21

**Authors:** Chaz Hyseni, Ryan C. Garrick

**Affiliations:** Department of Biology, University of Mississippi, University, MS 38677, USA; rgarrick@olemiss.edu

**Keywords:** biodiversity, biogeography, competitive exclusion, ecological niche model, molecular taxonomic identification, PCR-RFLP, *Reticulitermes*, saproxylic, species richness

## Abstract

In both managed and unmanaged forests, termites are functionally important members of the dead-wood-associated (saproxylic) insect community. However, little is known about regional-scale environmental drivers of geographic distributions of termite species, and how these environmental factors impact co-occurrence among congeneric species. Here we focus on the southern Appalachian Mountains—a well-known center of endemism for forest biota—and use Ecological Niche Modeling (ENM) to examine the distributions of three species of *Reticulitermes* termites (i.e., *R. flavipes*, *R. virginicus*, and *R. malletei*). To overcome deficiencies in public databases, ENMs were underpinned by field-collected high-resolution occurrence records coupled with molecular taxonomic species identification. Spatial overlap among areas of predicted occurrence of each species was mapped, and aspects of niche similarity were quantified. We also identified environmental factors that most strongly contribute to among-species differences in occupancy. Overall, we found that *R. flavipes* and *R. virginicus* showed significant niche divergence, which was primarily driven by dry-season precipitation. Also, all three species were most likely to co-occur in the mid-latitudes of the study area (i.e., northern Alabama and Georgia, eastern Tennessee and western North Carolina), which is an area of considerable topographic complexity. This work provides important baseline information for follow-up studies of local-scale drivers of these species’ distributions. It also identifies specific geographic areas where future assessments of the frequency of true syntopy vs. micro-allopatry, and associated interspecific competitive interactions, should be focused.

## 1. Introduction

### 1.1. the Southern Appalachian Mountains: A Center of Endemism for Forest Biota

The southern Appalachian Mountains, extending latitudinally from northeast Alabama to northwest Virginia, are some of the oldest uplands in North America. These mountains have been exposed and unglaciated for over 100 million years [1]. Steep altitudinal precipitation gradients, a complex heavily dissected topography, and a humid, temperate climate, have shaped southern Appalachian forests into some of the most diverse environments in the eastern United States. [2]. While deciduous oak-hickory forests dominate much of the mid-elevation landscape [2], high elevations (above 1400 m) support spruce-fir forests [3], whereas mesic coves support hemlock, and pines are commonly found at xeric low- to mid-elevations [4].

The southern Appalachian Mountains are incredibly rich in biodiversity [5]. The region is thought to have served as a major Pleistocene refuge for numerous species. Past climatic cycles have affected distributions of forest biota, resulting in major range shifts or local extinction. Following the Last Glacial Maximum (ca. 21,000 years ago), recolonization is thought to have occurred relatively rapidly, from 7000–16,000 years ago [6,7,8,9,10]. The southern Appalachian Mountains are a well-known center of endemism for salamanders and other amphibians [11,12]. However, there is increasing evidence of short-range endemism in other groups, including dead wood-associated forest invertebrates (e.g., millipedes [13,14], cockroaches [15,16], and centipedes [17]).

### 1.2. Subterranean Termites: Functionally Important Ecosystem Service Providers in Temperate Forests

Dead-wood-dependent (saproxylic) arthropods play critical roles in maintaining healthy, productive forests by contributing to the decomposition of fallen trees and thus driving nutrient cycling that affects organisms at all trophic levels [18,19,20,21,22]. Indeed, rotting logs may be one of the most stable, thermally buffered, above-ground microhabitats that exist in forests, and the decomposition process has successional stages, facilitated by wood-feeding and wood-boring invertebrates [21,23]. Termites are some of the first to colonize a rotting log, and through feeding and tunneling activities of the worker caste, the dead-wood substrate is modified by the creation of galleries. Once established, these facilitate colonization by larger wood-feeding invertebrates [24]. Ultimately, the ecosystem engineering activities of termites contribute to enhancing the internal heterogeneity of logs, making them habitable by a diverse array of saproxylic species.

Termites in the genus *Reticulitermes* (Blattodea: Rhinotermitidae) are broadly distributed across the eastern United States. Morphological separation of species is notoriously difficult [25], particularly given that only the worker caste can usually be readily sampled. To address this, we developed an efficient molecular assay (i.e., polymerase chain reaction (PCR) amplification of a short region of mitochondrial cytochrome oxidase subunit II (COII) gene, followed by screening of restriction-fragment-length polymorphism (RFLP) banding profiles [26]) that can be used to distinguish each of the five eastern United States species. In the southern Appalachians, several *Reticulitermes* species can co-occur locally. However, true syntopy (i.e., two species co-inhabiting the same rotting log) appears to be very rare, but reported instances of fine-scale sampling have been limited.

### 1.3. Ecological Niche Models: Efficient Tools for Predicting Organismal Distributions

Ecological niche models (ENMs) are broadly useful spatially explicit analytical tools that relate species occurrence data with environmental variables, such as climatic temperature and precipitation data [27], or topographic and land cover data. Once constructed, ENMs generate maps of estimated habitat suitability, and can be used to describe the historical, current, and future climate space for a given species. For example, ENMs have been used to identify areas of high conservation importance [28,29,30], predict climate change effects on geographic ranges of species [31,32], as well as determine potential threats of invasive species [33,34]. These analytical tools are becoming widely used owing to the increasing accessibility of climatic data via public databases [35,36,37]. An important assumption when using ENMs to predict historical or future distributions is niche conservatism (i.e., the stability of ecological niches over time) [38]. However, evidence suggests that niche conservatism is common among closely related species [39,40,41], and the risks of erroneous inferences are further reduced when focusing only on contemporary climate and occurrence data (i.e., when reconstructing present-day ENMs).

### 1.4. the Current State of Knowledge about Subterranean Termite Distributions, and Goals of this Study

There is a general lack of data on the natural distributions of termites in temperate forests, given that most research has focused on damage that termites cause to man-made wooden structures. Accordingly, occurrence records mostly come from urban areas, and they are also of low resolution (e.g., presence/absence in a given county). Notwithstanding these limitations, Maynard et al. [42] recently provided valuable insights into the role of climatic (temperature and precipitation) variables in influencing distributions of termites in the eastern United States. Specifically, those authors performed ENM for two *Reticulitermes* species (*R. flavipes* and *R. virginicus*) and the invasive Formosan subterranean termite, *Coptotermes formosanus*. Furthermore, they synthesized pre-existing knowledge to identify the influence on termite distributions of biotic factors, such as tree species and wood traits, fungal preferences, phenology of predatory ants, and competitive asymmetries among coexisting termite species. While interspecific competition may result in spatial or temporal separation which could lead to niche divergence, to date, very little is known about niche partitioning in subterranean termites and the environmental factors that may lead to niche divergence. 

In the present paper, we aimed to generate new insights into regional-scale environmental drivers of geographic distributions of termite species, and how these environmental factors impact co-occurrence among congeneric species. Focusing on the southern Appalachian Mountains and surrounding areas, we performed an ENM-based evaluation of niche divergence among the three most common *Reticulitermes* species in the eastern United States. In addition to identifying niche divergence, if present, we aimed to determine the environmental factors driving niche divergence among species.

## 2. Materials and Methods

### 2.1. Termite Sampling, Species Identification, and Ecological Niche Modeling

From 2012 to 2016, we collected *Reticulitermes* termites from 132 sites across the southern Appalachians Mountains and surrounding areas (Table S1; Figure S1). At most sites, termite workers were collected from a single rotting log at an intermediate to late stage of decay. However, at 10 sites, termites were also collected from additional logs within ~30 m of one another (i.e., samples came from a total of 2 logs at 8 sites, 3 logs at 1 site, and 4 logs at 1 site; Table S1). Owing to the close proximity of these clustered logs (i.e., at or near the typical error associated with a handheld GPS unit), the same coordinates were assigned to them, but specimen collections were assigned log-specific identifiers. Molecular taxonomic identifications were based on a single termite per rotting log, using Garrick et al.’s [26] PCR-RFLP assay. Briefly, a short (376-bp) region of the mitochondrial COII gene was amplified (using PCR primers RetCo2-F and RetCo2-R), and products were then sequentially digested with three restriction enzymes (RsaI, TaqI, and MspI), which in combination generate diagnostic species-specific banding patterns. Ultimately, we identified 91 non-redundant occurrence points for *R. flavipes*, 30 for *R. virginicus*, and 17 for *R. malletei* (Table S1). ENM was conducted with the ‘biomod2’ package [43] in R [44] using four modeling algorithms (e.g., [45,46,47]). Distributions were reconstructed using mean climatological data for a period spanning 1960–1990, with all variables used at 1-km resolution. Nineteen bioclimatic variables [35] were obtained from the WorldClim database v.1.4 (http://www.worldclim.org), and then factor analysis was used to reduce the number of predictors, and the associated correlation among them (see Files S1 for full details of ENM methods). From the 19 bioclimatic variables, we generated four environmental factors (see File S1 and Figures S2 and S3 for full details of factor analysis): dry-season precipitation, wet-season precipitation, summer temperature, and temperature range. 

### 2.2. Niche Occupancy, Niche Identity, and Distributional Overlap

Predicted niche occupancy profiles were generated for each environmental factor following Evans et al. [48], implemented in the ‘phyloclim’ package [49]. Niche overlap for each environmental factor was summarized using both Schoener’s D statistic [50], and the modified Hellinger statistic, I, as proposed by Warren et al. [51]. We also used the D and I statistics to determine pairwise niche equivalency/identity among the three *Reticulitermes* species. The niche equivalency test asks whether the ENMs of two species are more different than expected if they had been drawn from the same distribution. To perform the niche equivalency test, we generated a distribution using 999 pseudoreplicate datasets. 

To assess distributional overlap based on ENMs, we used maps of binary presence/absence as well as continuous occurrence probabilities. We used binary predictions, because this allowed us to determine which species co-occurred in areas of distributional overlap. However, since the use of continuous predictions has been recommended when estimating species richness [52], we calculated the sum of *Reticulitermes* species’ occurrence probabilities (Figure S4), and calculated joint and exclusive occurrence probabilities for each of the three species (Figure S5). For binary predictions, the approach of maximizing sensitivity and specificity has consistently performed better than other methods [53,54,55]. Thus, we used the True Skill Statistic (TSS = sensitivity + specificity − 1) [56] both as a model performance metric and to identify a threshold for converting continuous occurrence probabilities to binary classifications. The threshold was chosen based on maximizing the TSS, without risking under-prediction of presences (i.e., selecting the lowest threshold at which TSS is maximized). We used a threshold value of 0.2, where probability > 0.2 represented presence, and suitability ≤ 0.2 represented absence. We merged the three species’ binary maps by summing re-coded maps, where absence = 0, but presence was coded depending on species: *R. flavipes* = 4, *R. virginicus* = 2, and *R. malletei* = 1. This way, the sum of binary maps resulted in seven distinct categories: single-species areas (3 categories, with aforementioned scores); areas of two-species overlap (3 categories, scores of either 3, 5, or 6 depending on the identity of the species pair); and areas where all three species overlap (1 category, with a score of 7).

### 2.3. Environmental Factors and Niche Divergence

To determine the sources of variation in the *Reticulitermes* occurrence dataset, we included the effects of spatial structure and environmental factors, and performed variance partitioning using the ‘varpart’ function in ‘vegan’ [57]. To account for multiple predictors in the model, we used adjusted R^2^. To determine which (if any) environmental factors have significantly contributed to niche divergence of *Reticulitermes* species, we first removed the effect of spatial structure. We did this by performing distance-based redundancy analysis [58] using the ‘capscale’ function. To account for spatial structure, we transformed Euclidean geographic distances to a continuous rectangular vector by Principal Coordinates analysis of Neighbor Matrices (PCNM) using the ‘pcnm’ function in ‘vegan’. Only significant PCNM axes were used in partialling out spatial structure. Significance of the environmental and spatial predictors was assessed using multivariate F-statistics with 9999 permutations.

## 3. Results

### 3.1. Niche Occupancy, Niche Identity, and Distributional Overlap

**Figure 1:**
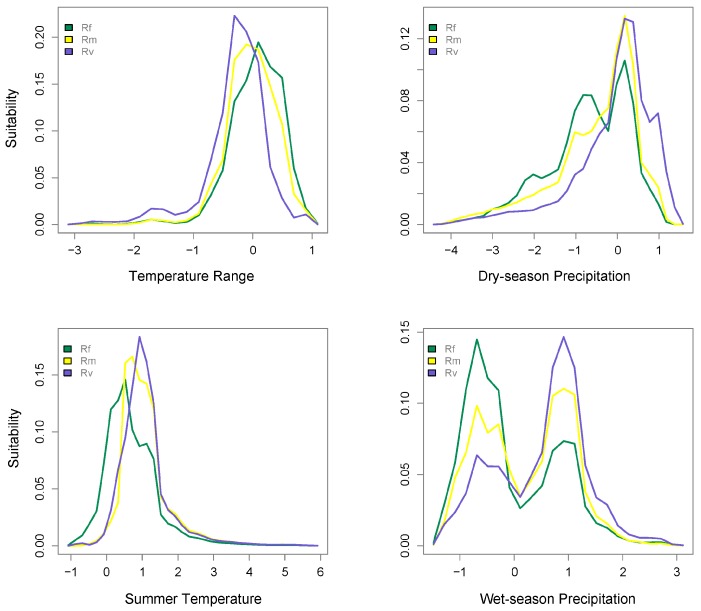
Predicted niche occupancy. Four environmental factors were used to estimate niche occupancy of *R. flavipes* (Rf), *R. malletei* (Rm), and *R. virginicus* (Rv): left two panels: temperature range and summer temperature; right two panels: dry- and wet-season precipitation. The y-axis represents niche occupancy, or suitability, and the area under the curves sums to 1, the total suitability.

Predicted niche occupancy profiles for the three *Reticulitermes* species (Figure 1) showed differences in peak values across all four environmental factors. The two temperature factors, summer temperature and temperature range, showed differences in peaks between *R. flavipes* and *R. virginicus*, whereas *R. malletei* was intermediate. Similarly, the two precipitation factors, dry-season precipitation and wet-season precipitation, showed more marked differences between *R. flavipes* and *R. virginicus* than for any of the other pairwise species comparisons. The bimodality of wet-season precipitation is a result of occurrence of *Reticulitermes* species in two areas with pronounced differences in wet-season precipitation (see Figure S3). Statistics that characterize the extent of niche overlap showed the least amount of niche overlap was between *R. flavipes* and *R. virginicus* (D = 0.582, I = 0.843; Table 1). Furthermore, the niche identity test between these two species showed significant differentiation (*p* < 0.001; Table 1). *R. malletei* was more similar to *R. flavipes* in terms of temperature range (D = 0.889) and dry-season precipitation (D = 0.872), but showed more overlap with *R. virginicus* for summer temperature (D = 0.894) and wet-season precipitation (D = 0.848). *R. virginicus* showed the least overlap with *R. flavipes*, across all four environmental factors (Table 2). 

**Figure 2:**
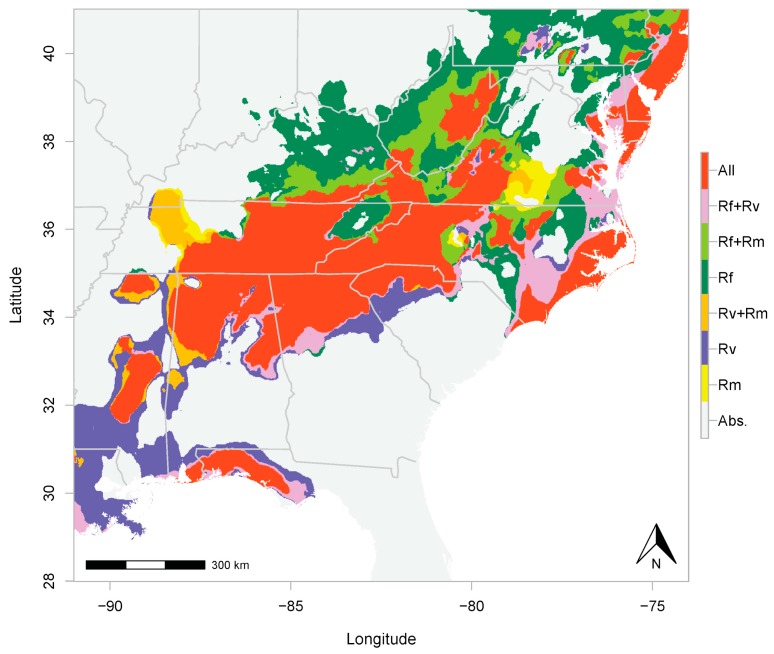
Distributional overlap of *R. flavipes* (Rf), *R. malletei* (Rm), and *R. virginicus* (Rv). Overlap is color coded based on the number of species. “All” is where occurrence of all three species is predicted. Areas of two-species overlap are shown in the legend as “Rf + Rv”, “Rf + Rm”, and “Rv + Rm”. Absence of all three species is shown in grey and referred to in the legend as “Abs.”

The predicted distribution of *R. flavipes* spanned a larger area in the northern portion of the southern Appalachians than that of the other two species. *R. flavipes* overlapped with *R. malletei*, to the exclusion of *R. virginicus*, in an area including Kentucky, Virginia, and West Virginia (Figure 2; Figure S5). The overlap between *R. flavipes* and *R. virginicus*, excluding *R. malletei*, spanned a smaller area, with lower probability (Figure S5). Predicted distributions of all three species overlapped in eastern Tennessee, western North Carolina, northern Alabama and Georgia (Figure 2; Figures S4 and S5). 

### 3.2. Environmental Factors and Niche Divergence

**Figure 3:**
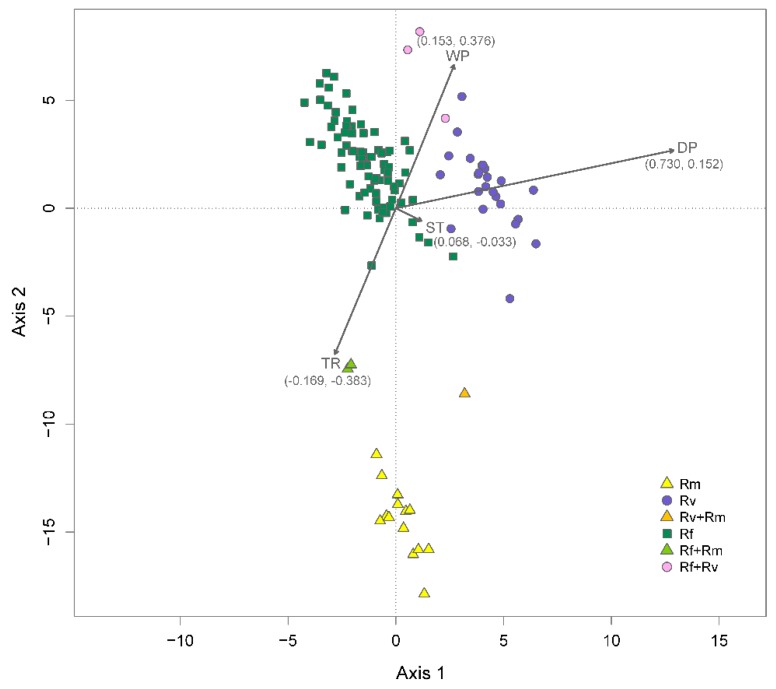
Distance-based redundancy analysis. The plot shows a constrained ordination of 132 sampling sites, color coded based on the number of species present. Sites where only *R. flavipes*, *R. virginicus*, or *R. malletei* were sampled are referred to in the legend as “Rf”, “Rv”, and “Rm”, respectively. Two-species sites are shown in the legend as “Rf + Rv”, “Rf + Rm”, and “Rv + Rm”. The ordination is conditional on six significant spatial components (PCNM axes 1, 4, 6, 17, 43, and 58) and constrained by four environmental factors: dry-season precipitation (DP); wet-season precipitation (WP); summer temperature (ST); temperature range (TR). Arrows show strength of correlation (coefficients in parentheses) of environmental factors with ordination axes 1 and 2.

Distance-based redundancy analysis (Figure 3) showed that only the dry-season precipitation factor contributed significantly (F_1, 127_ = 8.673, *p* = 0.001) to differences in occurrence among the three *Reticulitermes* species. After accounting for spatial structure by partialling out six significant spatial components (PCNM axes 1, 4, 6, 17, 43, and 58), dry-season precipitation remained significant (F_1, 121_ = 5.622, *p* = 0.003). The six significant spatial components along with dry-season precipitation accounted for 18.5% of the observed variation in the occurrence data. Spatial structure alone explained 9.6% of the variation, environmental factors accounted for 3.3%, and the interaction between the two explained an additional 5.6% of the variation. 

Following the removal of spatial structure effects, the highest correlation coefficient between environmental factors and ordination axes of the distance-based redundancy analysis was observed for dry-season precipitation (r = 0.730) and axis 1. This axis captured the divergence of *R. virginicus* from the other two species (Figure 3). Thus, dry-season precipitation contributed significantly to *R. virginicus* divergence. While not significant, temperature range (r = −0.383) and wet-quarter precipitation (r = 0.376) were correlated with axis 2, which captured the divergence of *R. malletei* (Figure 3).

## 4. Discussion

This study provides insights into the ecology of subterranean termites with regard to geographic distributions and niche partitioning among three broadly co-distributed *Reticulitermes* species in the southern Appalachians Mountains and surrounding areas. This region is a biogeographically significant center of endemism, yet the ecology of its resident invertebrate fauna—particularly saproxylic insects—is poorly known. Our ENMs suggest that an area in the mid-latitudes of the southern Appalachians, characterized by complex topography and multiple ecoregions, provides suitable habitat to support all three *Reticulitermes* species. Our study also highlights the roles that temperature and precipitation play in driving niche divergence among *Reticulitermes* species. To our knowledge, this work represents the first evidence of significant regional-scale niche divergence between *R. flavipes* and *R. virginicus*. Below, we consider the broader context of these findings, as well as caveats and future directions for follow-up studies that build on the information presented here.

### 4.1. *Reticulitermes* Distributions and Climatic Drivers of Niche Divergence among Species

Our analyses predicted extensive co-occurrence of all three *Reticulitermes* species in the mid-latitudes of the southern Appalachians (Figure 2; Figures S4 and S5). Based on paleoclimatic [59], biogeographic [60] and comparative phylogeographic [61] data, the southern Appalachians remained free from Pleistocene ice sheets and served as a major refuge for many species during glacial periods, consequently maintaining higher levels of biodiversity. Indeed, the present-day complexity of this mid-latitude region harbors many different niches, which could facilitate long-term coexistence of closely related species. However, in addition to predicted co-occurrence of *Reticulitermes* species in the montane regions of the southern Appalachians, our ENMs also identified areas of two- and three-species co-occurrence along the Gulf coast of western Florida, and the Atlantic coast from North Carolina to New Jersey and New York. To empirically confirm the co-occurrence of subterranean termites in these coastal areas, future studies should include these regions in their sampling efforts. In the case of another forest-dependent invertebrate, the millipede *Narceus americanus*, the Florida Gulf coast has been identified as an important refuge during the Last Glacial Maximum [62]. Indeed, the paleoclimatic history of areas to the south and east of the southern Appalachian Mountains are increasingly being recognized as reservoirs of forest invertebrate biodiversity during past periods of environment change. The incidence of high termite species diversity—even though only assessed here for one genus—is therefore not unexpected.

In addition to co-occurrence of *Reticulitermes* species, our study provides novel insights into climatic drivers of niche divergence. Consistent with the findings of Maynard et al. [42], we determined that *R. virginicus* is more restricted to the south, whereas *R. flavipes* has a broad latitudinal range. Furthermore, we determined that *R. flavipes* occurs farther north than the other two species, even excluding other *Reticulitermes* (Figure S5), potentially because it tolerates lower amounts of precipitation (both dry- and wet-season; Figure 1). Maynard et al.’s [42] ENMs showed that temperature variables were the most important predictors of termite distributions. Based on our formal assessment of niche overlap between *R. flavipes* and *R. virginicus*, we determined that both temperature and precipitation seasonality (as represented by temperature range, summer temperature, and dry- and wet-season precipitation) play non-negligible roles in the significant niche divergence between *R. flavipes* and *R. virginicus*. Furthermore, using distance-based redundancy analysis, we identified dry-season precipitation as a major driver of this divergence. In the mid-latitudes of the southern Appalachians, where dry-season precipitation is high (Figure S3), all three *Reticulitermes* species co-occur (Figure 2; Figures S4 and S5), but farther north, where dry- and wet-season precipitation is low (Figure S3), *R. flavipes* is more competitive.

### 4.2. Potential Explanations for Lack of Empirical Evidence for Local-Scale Coexistence of *Reticulitermes* Species

Interestingly, despite the significant niche divergence between *R. flavipes* and *R. virginicus*, we collected both of these species from the same rotting log at one sampling site (i.e., #37 located near the Georgia/Southern Carolina state border; Table S1). To our knowledge, this is the first record of true syntopy between *Reticulitermes* species. The apparent rarity of syntopy and general lack of coexistence of *Reticulitermes* species at local scales could be explained by competitive exclusion. Colony size and soldier number are important features for termite competitive ability. Termite species with small colonies have been observed to relinquish resources and be eliminated by dominant interspecific competitors with large colonies [63]. Through avoidance of dominant competitors, interspecific competition may result in spatial separation [64], but also temporal separation (i.e., phenological differences). Termites may be able to avoid other related species using vibrational cues. Indeed, vibrational cues are important for termite sensory perception and communication, as these signals can travel over long distances [65,66]. For instance, the drywood termite *Cryptotermes secundus* can distinguish conspecifics from the dominant competitor in the environment, the subterranean termite *Coptotermes acinaciformis* [65]. Furthermore, *Coptotermes acinaciformis* detects its major predator, the ant *Iridomyrmex purpureus*, using vibrational cues only [66]. Overall, given these highly tuned sensory capabilities, it stands to reason that competitive exclusion, or competitor avoidance, could be important factors in preventing local co-occurrence among *Reticulitermes* species. Alternatively, the dominant competitor may ultimately outcompete the other species. For instance, *R. flavipes* has a broad distribution and occurs farther north than the other two species, possibly due to a competitive advantage stemming from the fact that it tolerates conditions of lower dry- and wet-season precipitation. Furthermore, interspecific aggression coupled with low levels of intraspecific agonism (even colony fusion) [67,68], may make *R. flavipes* the dominant competitor.

### 4.3. Caveats and Future Directions

While our sampling suggests that true syntopy and local co-occurrence of different species at the same site is very rare, our detection of only one species in all but one rotting log, and at the majority of sampling sites (i.e., 126 out of 132), may actually be a consequence of the sampling strategy that was employed (see Section 2.1). Briefly, we simply aimed to collect termites from each site, rather than provide a complete assessment of termite diversity at each site. Indeed, variance partitioning reflects this, showing that most (81.5%) of the variance in the occurrence data did not stem from spatial structure (9.6%), or environmental differences (3.3%), or interaction between the two (5.6%). Accordingly, while competitive exclusion is a plausible explanation for apparent rare local-scale co-occurrence (i.e., micro-allopatry) among *Reticulitermes* species, a dedicated sampling approach would be required to formally test this idea. For example, exhaustively sampling multiple logs per site, at a series of sites arranged along a transect traversing a region where two or more species occur in close proximity would be a productive approach. Fortunately, the present study identified specific geographic areas where future assessments of the frequency of true syntopy vs. micro-allopatry, and associated interspecific competitive interactions, should be focused (Table S1; Figure S1).

Although we have shown separation in niche space between species, particularly *R. flavipes* and *R. virginicus*, these inferences were underpinned by regional-scale environmental variables, and so they do not take into account local-scale drivers of niche divergence such as differences in microhabitat preference, phenology, or diet. Indeed, Maynard et al. [42] highlighted that biotic and soil characteristics play a role in termite distribution and abundance. Thus, our assessment of niche divergence is necessarily incomplete. While it does provide important baseline information, follow-up studies of local-scale drivers of species’ distributions could examine aspects of the microhabitat (e.g., humidity and temperature of soil and rotting logs), timing of nuptial flights along latitudinal and altitudinal clines, and/or use stable isotopes to determine decomposition stage of ingested wood and the importance of microbial biomass in termite diets at a given location [69].

## Figures and Tables

**Table 1:** Niche identity test. The upper off-diagonal shows Schoener’s D statistic, and the lower off-diagonals shows the modified Hellinger statistic, I. Significant niche divergence is reported in bold text with red highlighting. The more dissimilar of the other two niche comparisons is highlighted in pink. Abbreviations used for *R. flavipes*, *R. malletei*, and *R. virginicus* are Rf, Rm, and Rv, respectively.

	Rf	Rm	Rv
Rf	-	D = 0.744 *p* = 0.280	**D = 0.582** ***p* < 0.001**
Rm	I = 0.935 *p* = 0.239	-	D = 0.788 *p* = 0.630
Rv	**I = 0.843** ***p* < 0.001**	I = 0.961 *p* = 0.750	-

**Table 2:** Pairwise niche overlap among *Reticulitermes* species for each of four environmental factors. The top three rows show Schoener’s D statistic, and the bottom three rows show the modified Hellinger statistic, I. The four environmental factors are: temperature range (TR), dry-season precipitation (DP), summer temperature (ST), and wet-season precipitation (WP). Niche overlap is highest in green and lowest in red. *R. flavipes*, *R. malletei*, and *R. virginicus* are abbreviated as Rf, Rm, and Rv, respectively.

		TR	DP	ST	WP
**D**	Rf/Rm	0.889	0.872	0.693	0.820
	Rf/Rv	0.683	0.707	0.680	0.680
	Rm/Rv	0.791	0.809	0.894	0.848
**I**	Rf/Rm	0.991	0.990	0.919	0.982
	Rf/Rv	0.917	0.928	0.926	0.942
	Rm/Rv	0.952	0.961	0.990	0.984

## Supplementary Materials

The following are available online at http://www.mdpi.com/2075-4450/10/1/33/s1, File S1: Environmental variables and Ecological Niche Modeling methods; Table S1: Sampling sites with number of species occurrences at each site and number of logs per site; Figure S1: Map of *Reticulitermes* sampling depicting occurrences of one or more species at each site; Figure S2: Factor analysis; Figure S3: Environmental factors and bioclimatic variables; Figure S4: Distributional overlap of *Reticulitermes* species; Figure S5: Probability of joint and exclusive occurrence of *Reticulitermes* species.
