# Correction: Hyseni, C.; Garrick, R.C. Ecological Drivers of Species Distributions and Niche Overlap for Three Subterranean Termite Species in the Southern Appalachian Mountains, USA. *Insects* **2019**, *10*, 33

**DOI:** 10.3390/insects11030148

**Published:** 2020-02-26

**Authors:** Chaz Hyseni, Ryan C. Garrick

**Affiliations:** Department of Biology, University of Mississippi, University, MS 38677, USA; rgarrick@olemiss.edu

It has recently come to our attention that two of the environmental rasters we used for analyses in our study [1] were mislabeled in a raster processing pipeline. As a result, in the article [1], all references to dry-season precipitation and summer temperature require switching. Here, Figure 1 and Figure 3 and Table 2, are the corrected versions of Figures 1 and 3 and Table 2 in the article [1].

In the abstract, one of the sentences should read: “Overall, we found that *R. flavipes* and *R. virginicus* showed significant niche divergence, which was primarily driven by summer temperature.”

The first paragraph in the Results section should read: “Bimodality was also observed for summer temperature in *R. flavipes*, given that the species occurs in both low elevations and the cooler high-elevation areas of the Appalachians (see Figure S3). Statistics that characterize the extent of niche overlap showed that *R. flavipes* and *R. virginicus* had the least amount of overlap (D = 0.582, I = 0.843).” In the same paragraph, a correction is required two sentences later: “*R. malletei* was more similar to *R. flavipes* in terms of temperature range (D = 0.889) and summer temperature (D = 0.872) but showed more overlap with *R. virginicus* for dry- (D = 0.894) and wet-season precipitation (D = 0.848).”

In the “Environmental Factors and Niche Divergence” subsection of the Results, all references to dry-season precipitation should actually be references to summer temperature. The last correction is in the Discussion section, in the last paragraph of the “*Reticulitermes* Distributions and Climatic Drivers of Niche Divergence among Species” subsection: “Furthermore, using distance-based redundancy analysis, we identified summer temperature as a major driver of this divergence.”

The authors wish to apologize for the error and any inconvenience this may have caused.

## Figures and Tables

**Figure 1:**
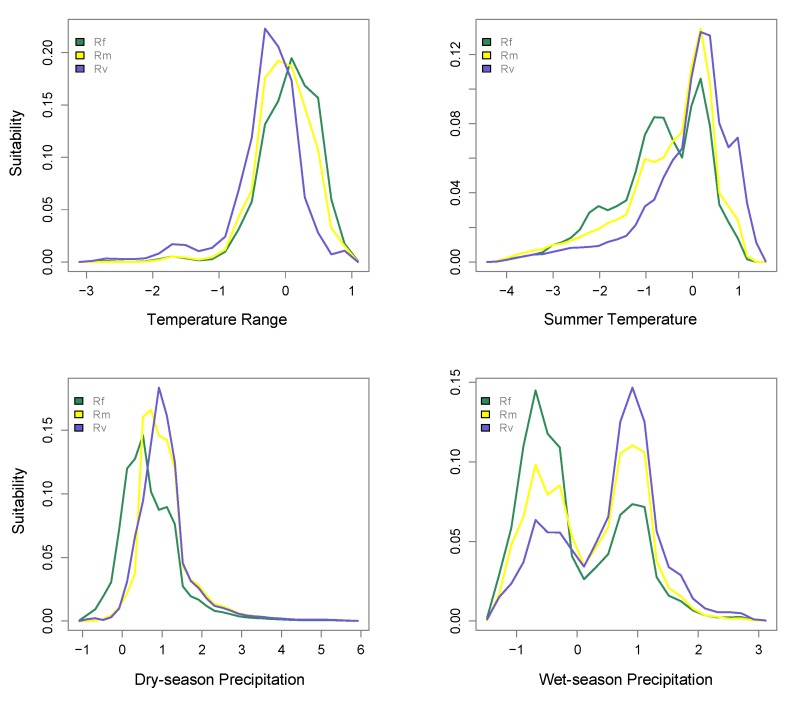
Predicted niche occupancy. Four environmental factors were used to estimate niche occupancy of *R. flavipes* (Rf), *R. malletei* (Rm), and *R. virginicus* (Rv): top two panels: temperature range and summer temperature; bottom two panels: dry- and wet-season precipitation. The *y*-axis represents niche occupancy, or suitability, and the area under the curves sums to 1, the total suitability.

**Figure 3:**
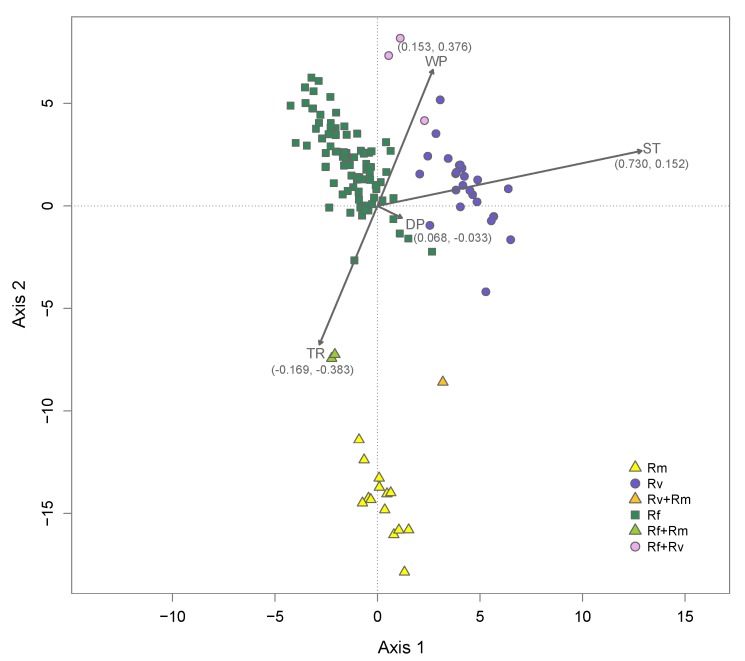
Distance-based redundancy analysis. The plot shows a constrained ordination of 132 sampling sites, color-coded based on the number of species present. Sites where only *R. flavipes*, *R. virginicus*, or *R. malletei* were sampled are referred to in the legend as “Rf”, “Rv”, and “Rm”, respectively. Two-species sites are shown in the legend as “Rf + Rv”, “Rf + Rm”, and “Rv + Rm”. The ordination is conditional on six significant spatial components (PCNM axes 1, 4, 6, 17, 43, and 58) and constrained by four environmental factors: dry-season precipitation (DP); wet-season precipitation (WP); summer temperature (ST); temperature range (TR). Arrows show strength of correlation (coefficients in parentheses) of environmental factors with ordination axes 1 and 2.

**Table 2:** Pairwise niche overlap among *Reticulitermes* species for each of four environmental factors. The top three rows show Schoener’s D statistic, and the bottom three rows show the modified Hellinger statistic, I. The four environmental factors are: temperature range (TR), summer temperature (ST), dry-season precipitation (DP), and wet-season precipitation (WP). Niche overlap is highest in green and lowest in red. *R. flavipes*, *R. malletei*, and *R. virginicus* are abbreviated as Rf, Rm, and Rv, respectively.

		TR	ST	DP	WP
	Rf/Rm	0.889	0.872	0.693	0.820
**D**	Rf/Rv	0.683	0.707	0.680	0.680
	Rm/Rv	0.791	0.809	0.894	0.848
	Rf/Rm	0.991	0.990	0.919	0.982
**I**	Rf/Rv	0.917	0.928	0.926	0.942
	Rm/Rv	0.952	0.961	0.990	0.984
