# Supplementary material for: Ecological Drivers of Species Distributions and Niche Overlap for Three Subterranean Termite Species in the Southern Appalachian Mountains, USA
Source: Insects. 2019 Jan 21;10(1):33. doi: 10.3390/insects10010033 (PMC6359368; doi:10.3390/insects10010033)
Supplement: Supplementary file 1 [file insects-10-00033-s001.zip › SUPPLY/Figure S1.docx]

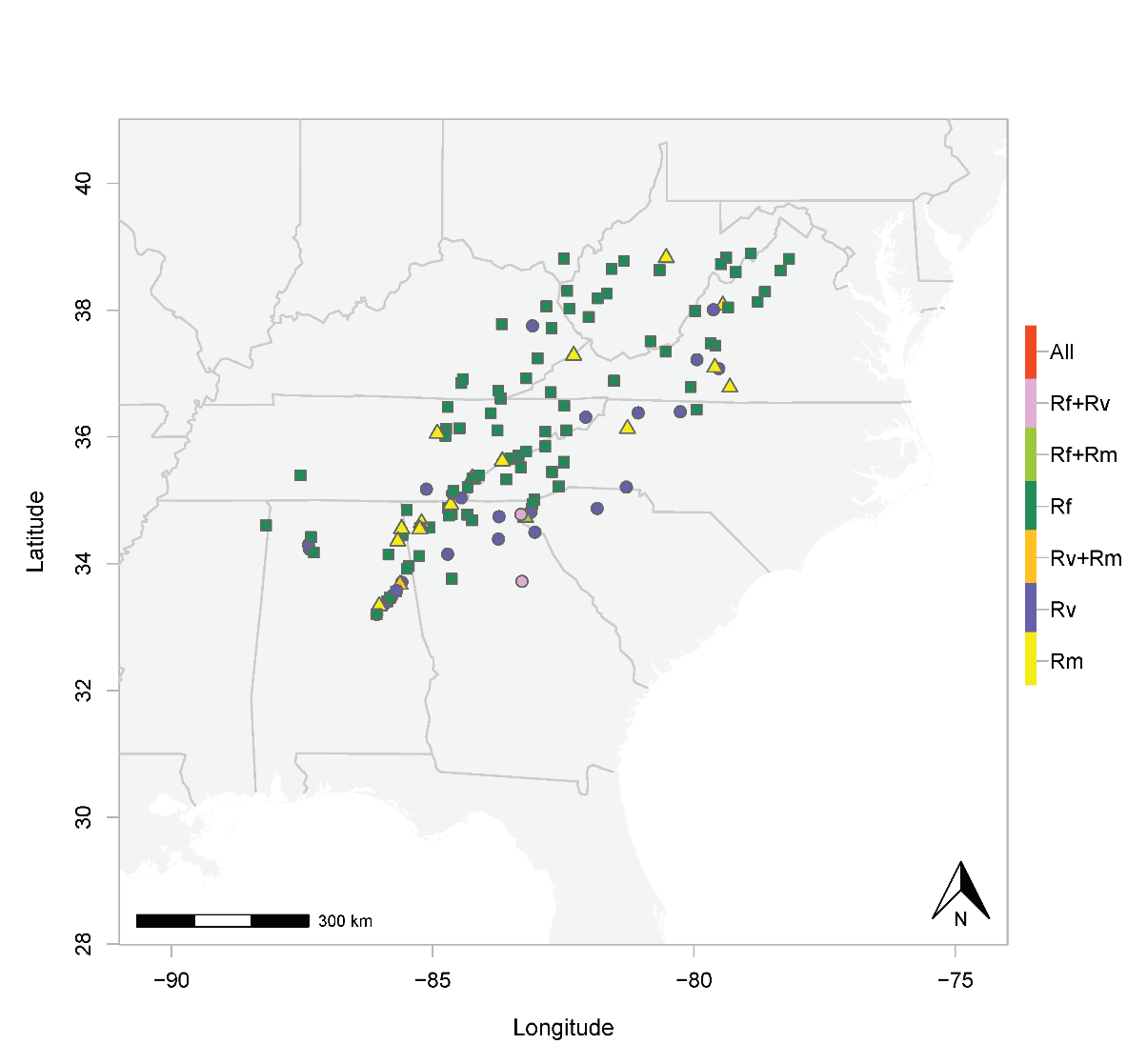


**Figure S1.** Map of Reticulitermes sampling depicting occurrences of one or more species at each site. Abbreviations used for *R. flavipes*, *R. malletei*, and *R. virginicus* are Rf, Rm, and Rv, respectively. Sites are color coded based on the number of species detected. There were no sites with all three species (“All”). The sites with two species are shown in the legend as “Rf + Rv,” “Rf + Rm,” and “Rv + Rm.”.
