# Supplementary material for: Ecological Drivers of Species Distributions and Niche Overlap for Three Subterranean Termite Species in the Southern Appalachian Mountains, USA
Source: Insects. 2019 Jan 21;10(1):33. doi: 10.3390/insects10010033 (PMC6359368; doi:10.3390/insects10010033)
Supplement: Supplementary file 1 [file insects-10-00033-s001.zip › SUPPLY/Figure S2.docx]

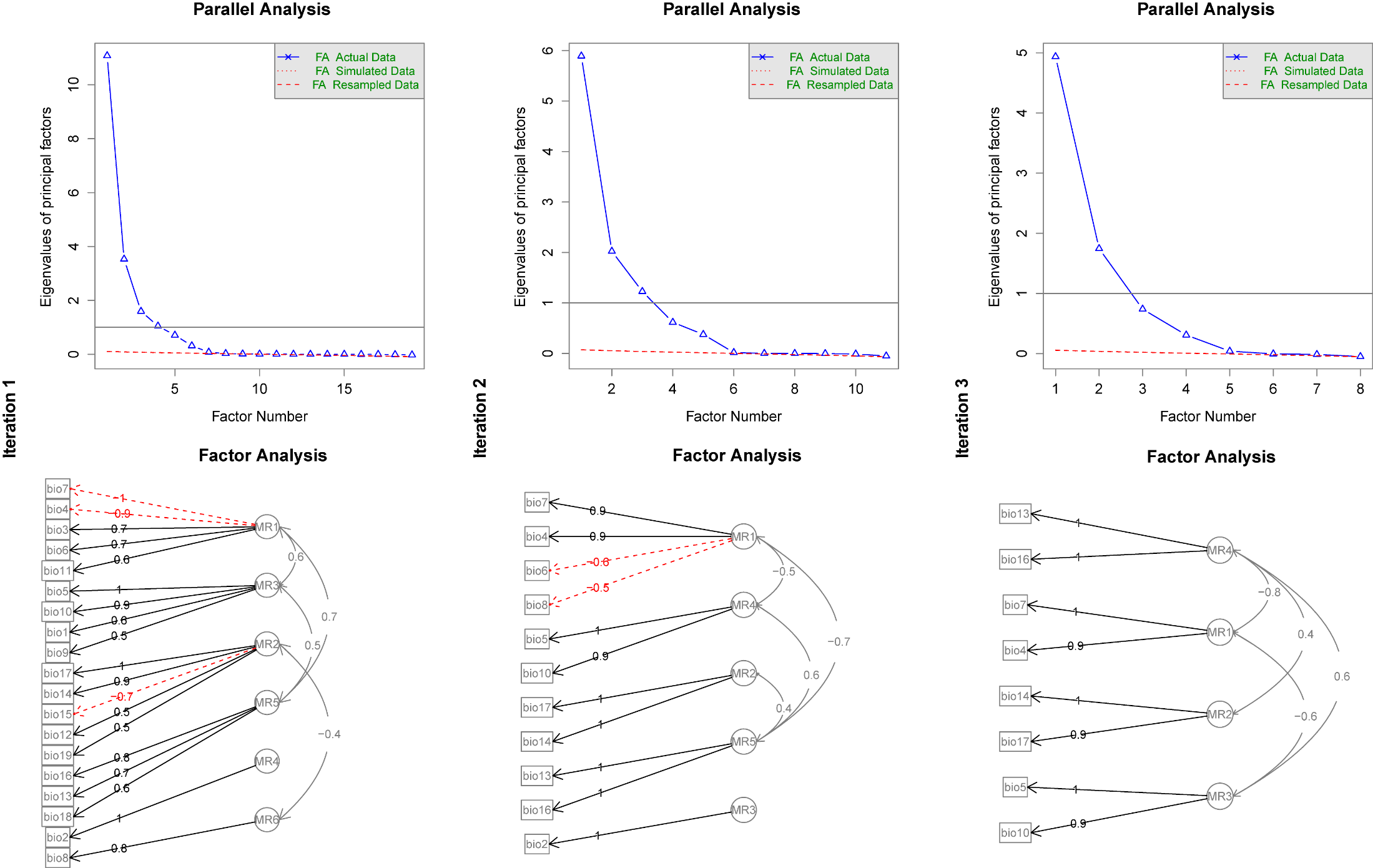


**Figure S2.** Factor analysis. Each column of panels represents one of three iterations of factor analysis. The top row depicts scree plots showing eigenvalues in descending order, the traditional threshold where eigenvalue = 1, and the confidence interval (red dotted lines) obtained via parallel analysis. The bottom row shows the factors and strength of correlation with the original bioclimatic variables. In the third and final iteration, abbreviations are as follows: MR1: temperature range; MR2: dry-season precipitation; MR3: summer temperature; MR4: wet-season precipitation.
