# Supplementary material for: Ecological Drivers of Species Distributions and Niche Overlap for Three Subterranean Termite Species in the Southern Appalachian Mountains, USA
Source: Insects. 2019 Jan 21;10(1):33. doi: 10.3390/insects10010033 (PMC6359368; doi:10.3390/insects10010033)
Supplement: Supplementary file 1 [file insects-10-00033-s001.zip › SUPPLY/Figure S3.docx]

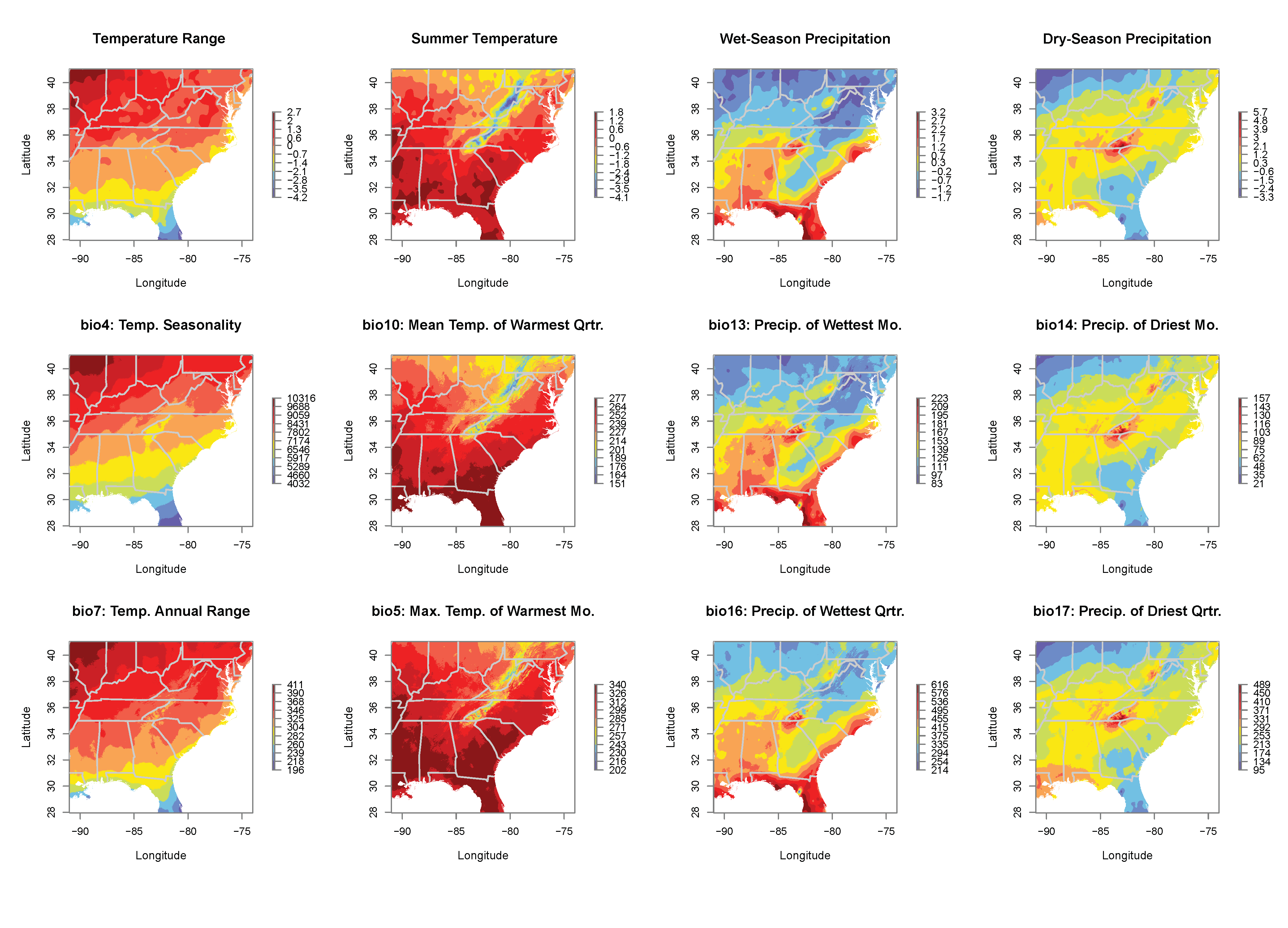


**Figure S3.** Environmental factors and bioclimatic variables. The top row of panels shows the four environmental factors obtained via factor analysis (see Figure S2). In each column of panels, the top panel shows the factor that explains the variation in the original bioclimatic variables, whereas the middle and bottom panels show the bioclimatic variables that correlate most strongly with the factor in the top panel. Note that the scales are different for each panel, but the colors go from dark blue (lowest value) to dark red (highest value). The environmental factors are unitless and go from negative to positive values. The unit for temperature variables is °C x 10. The unit for precipitation variables is mm.
