# Supplementary material for: Ecological Drivers of Species Distributions and Niche Overlap for Three Subterranean Termite Species in the Southern Appalachian Mountains, USA
Source: Insects. 2019 Jan 21;10(1):33. doi: 10.3390/insects10010033 (PMC6359368; doi:10.3390/insects10010033)
Supplement: Supplementary file 1 [file insects-10-00033-s001.zip › SUPPLY/Figure S4.docx]

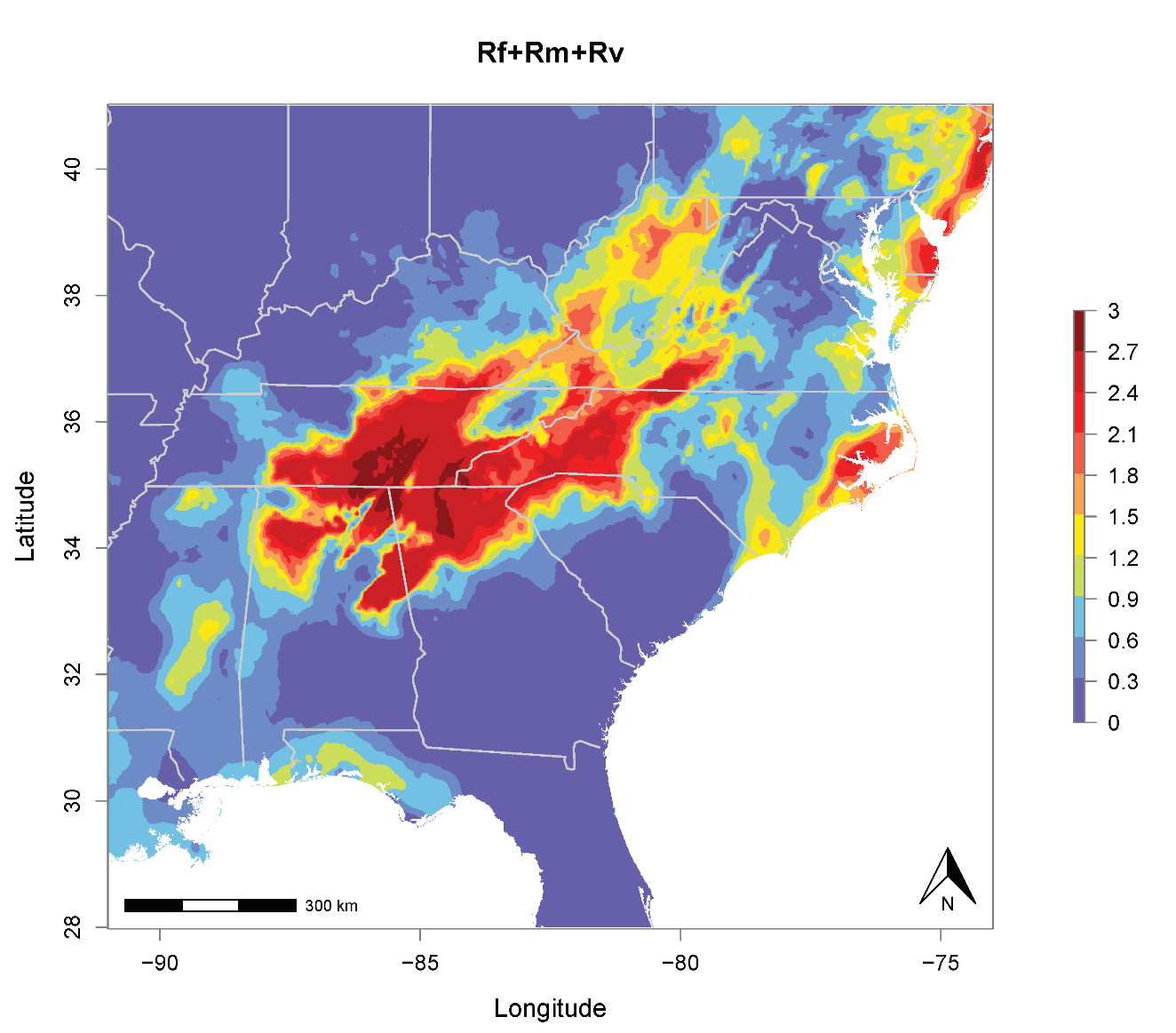


**Figure S4.** Distributional overlap of *Reticulitermes* species. Overlap is depicted based on the sum of individual species’ occurrence probabilities, with the highest value being 3 (dark red), where all three species co-occur at a probability of 1. Areas with occurrence probability above 1 (green to red) must have more than one species. Areas with probability below 1 (blues) could have more than one species with probabilities lower than 0.5. Absence of all three species is shown in dark blue.
