# Supplementary material for: Ecological Drivers of Species Distributions and Niche Overlap for Three Subterranean Termite Species in the Southern Appalachian Mountains, USA
Source: Insects. 2019 Jan 21;10(1):33. doi: 10.3390/insects10010033 (PMC6359368; doi:10.3390/insects10010033)
Supplement: Supplementary file 1 [file insects-10-00033-s001.zip › SUPPLY/Figure S5.docx]

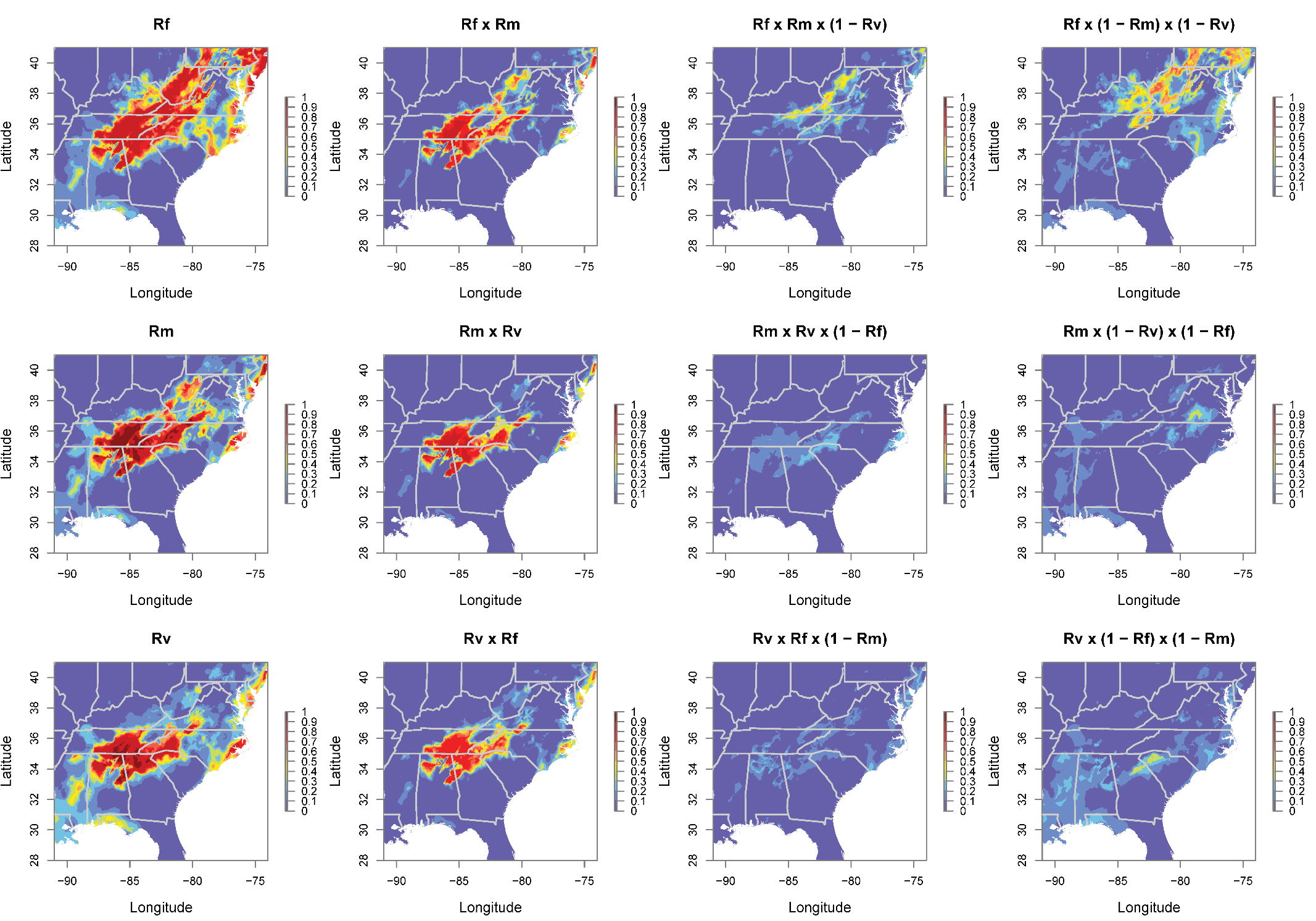


**Figure S5.** Probability of joint and exclusive occurrence of *Reticuliterme*s species. The leftmost column of panels shows probability of occurrence of *R. flavipes*, *R. malletei*, and *R. virginicus* (abbreviated as Rf, Rm, and Rv, respectively), whereas probability of absence is denoted as (1 – Rf), (1 – Rm), and (1 – Rv). Probability of occurrence is shown on a scale from 0 (dark blue) to 1 (dark red). The second column of panels shows probability of joint occurrence of two species (without excluding the third), expressed as products: “Rf x Rv,” “Rf x Rm,” and “Rv x Rm.” The third column shows areas where two species co-occur, but the third species is absent (probability of absence: 1 – Rf, 1 – Rm, 1 – Rv). Probability of occurrence of a single species, while excluding the other two, is shown in the rightmost column.
