# Supplementary material for: Ecological Drivers of Species Distributions and Niche Overlap for Three Subterranean Termite Species in the Southern Appalachian Mountains, USA
Source: Insects. 2019 Jan 21;10(1):33. doi: 10.3390/insects10010033 (PMC6359368; doi:10.3390/insects10010033)
Supplement: Supplementary file 1 [file insects-10-00033-s001.zip › SUPPLY/File S1.docx]

*Environmental variables and Ecological Niche Modeling methods*

Ecological Niche Models (ENMs) were constructed using the ‘biomod2’ package [1] in R [2]. To construct ENMs, in addition to presence records, we used pseudo-absence points, selected following Barbet-Massin et al. [3]. To do this, we first ran a rectilinear surface range envelope model [1], and then, from outside the area predicted as suitable habitat, we picked 100 random points. We created 20 independent sets of pseudo-absences, each of which were combined with the same 91 presence records. Four modeling algorithms were run: artificial neural networks [4], generalized boosted models or boosted regression trees [5], random forest [6], and maximum entropy [7]. We used 5 cross-validation runs per algorithm, for a total of 400 runs (4 algorithms x 5 cross-validations x 20 datasets), with 5,000 iterations per run. To assess model performance, 75% of the data were used for training, with 25% set aside as “out-of-bag” test data. To maximize the accuracy of presence/absence classification, we used the True Skill Statistic (TSS = sum of sensitivity and specificity – 1) [8], where ENMs with mean TSS above 0.2 were retained. We then used the ensemble framework [9] to obtain a weighted average of all ENMs, where ENMs were weighted according to TSS values.

Nineteen bioclimatic variables [10] were obtained from the WorldClim database v.1.4 (http://www.worldclim.org). To reduce the number of predictors, and correlation among them, we performed factor analysis in successive stages using the ‘psych’ package [11], until two criteria were met: 1) each factor must be highly correlated (absolute value of r > 0.5) with at least two variables, and 2) each variable must be highly correlated with only one factor and show low correlation (absolute value of r < 0.3) with any other factor. We used ordinary least squares to find the minimum residual (MR) solution [12]. Oblique rotations were used, since strong correlations between factors were expected. Cattell’s [13] scree test and Horn’s [14] parallel analysis determined the number of factors to retain, and these were then inspected for reliability using Cronbach’s [15] α, with an acceptance criterion of α > 0.7. The factors were named according to the bioclimatic variables they were most strongly correlated with. “Temperature Range” (TR; strongly correlated with bio4: “Temperature Seasonality” and bio7: “Temperature Annual Range”); “Dry-season Precipitation” (DP; strongly correlated with bio14: “Precipitation of Driest Month” and bio17: “Precipitation of Driest Quarter”); “Summer Temperature” (ST; strongly correlated with bio5: “Maximum Temperature of Warmest Month” and bio10: “Mean Temperature of Warmest Quarter”); “Wet-season Precipitation” (WP; strongly correlated with bio13: “Precipitation of Wettest Month” and bio17: “Precipitation of Wettest Quarter”).

References

1. Thuiller, W.; Lafourcade, B.; Engler, R.; Araújo, M.B. BIOMOD - a platform for ensemble forecasting of species distributions. *Ecography* **2009**, *32*, 369–373.
2. R Core Team. *R: A Language and Environment for Statistical Computing*. R Foundation for Statistical Computing, Vienna, Austria, 2018.
3. Barbet-Massin, M.; Jiguet, F.; Albert, C.H.; Thuiller, W. Selecting pseudo-absences for species distribution models: how, where and how many? *Methods Ecol. Evol.* **2012**, *3*, 327–338.
4. Ripley, B.D. *Pattern recognition and neural networks*; Cambridge University Press, 1996.
5. Friedman, J.H. Greedy function approximation: A gradient boosting machine. *Ann. Stat.* **2001**, *29*, 1189–1232.
6. Breiman, L. Random forests. *Mach. Learn.* **2001**, *45*, 5–32.
7. Phillips, S.J.; Anderson, R.P.; Schapire, R.E. Maximum entropy modeling of species geographic distributions. *Ecol. Modell.* **2006**, *190*, 231–259.
8. Allouche, O.; Tsoar, A.; Kadmon, R. Assessing the accuracy of species distribution models: prevalence, kappa and the true skill statistic (TSS). *J. Appl. Ecol.* **2006**, *43*, 1223–1232.
9. Buisson, L.; Thuiller, W.; Casajus, N.; Lek, S.; Grenouillet, G. Uncertainty in ensemble forecasting of species distribution. *Glob. Chang. Biol.* **2010**, *16*, 1145–1157.
10. Hijmans, R.J.; Cameron, S.E.; Parra, J.L.; Jones, P.G.; Jarvis, A. Very high resolution interpolated climate surfaces for global land areas. *Int. J. Climatol.* **2005**, *25*, 1965–1978.
11. Revelle, W. *psych: procedures for personality and psychological research*, 2018. R package version 1.8.10.
12. Harman, H.H.; Jones, W.H. Factor analysis by minimizing residuals (minres). *Psychometrika* **1966**, *31*, 351–368.
13. Cattell, R.B. The scree test for the number of factors. *Multivariate Behav. Res.* **1966**, *1*, 245–276.
14. Horn, J.L. A rationale and test for the number of factors in factor analysis. *Psychometrika* **1965***, 30*, 179–185.
15. Cronbach, L.J. Coefficient alpha and the internal structure of tests. *Psychometrika* **1951**, *16*, 297–334.
