# Supplementary material for: Ecological Drivers of Species Distributions and Niche Overlap for Three Subterranean Termite Species in the Southern Appalachian Mountains, USA
Source: Insects. 2019 Jan 21;10(1):33. doi: 10.3390/insects10010033 (PMC6359368; doi:10.3390/insects10010033)
Supplement: Supplementary file 1 [file insects-10-00033-s001.zip › SUPPLY/Table S1.docx]

**Table S1.** *Sampling sites with number of species occurrences at each site and number of logs per site*. Geographic coordinates and altitude (alt.) in meters for each site are reported. *R. flavipes*, *R. malletei*, and *R. virginicus* are abbreviated as Rf, Rm, and Rv, respectively. The number (#) of logs refers the number of logs sampled, from which termites were collected and identified to species (note that site 37 is the only site where two species were detected in the same log). Only non−redundant occurrence records were used for subsequent analyses.

| **Site** | **Longitude** | **Latitude** | **Alt. (m)** | **Rf** | **Rm** | **Rv** | **# of Logs** |
| --- | --- | --- | --- | --- | --- | --- | --- |
| 1 | −84.63805 | 34.77972 | 764 | 1 | 0 | 0 | 1 |
| 2 | −85.06536 | 34.57297 | 450 | 1 | 0 | 0 | 1 |
| 3 | −85.21630 | 34.64336 | 386 | 0 | 1 | 0 | 1 |
| 4 | −85.24268 | 34.56515 | 408 | 1 | 0 | 0 | 1 |
| 5 | −85.24043 | 34.56416 | 427 | 0 | 0 | 1 | 1 |
| 6 | −79.38618 | 38.82374 | 528 | 2 | 0 | 0 | 2 |
| 7 | −79.38506 | 38.82585 | 548 | 1 | 0 | 0 | 1 |
| 8 | −79.48494 | 38.72694 | 936 | 1 | 0 | 0 | 1 |
| 9 | −85.25067 | 34.54107 | 341 | 0 | 2 | 0 | 2 |
| 10 | −86.07185 | 33.20099 | 301 | 0 | 0 | 2 | 2 |
| 11 | −85.80658 | 33.47105 | 621 | 1 | 0 | 1 | 2 |
| 12 | −85.77732 | 33.49199 | 413 | 0 | 0 | 1 | 1 |
| 13 | −85.69289 | 33.57288 | 340 | 0 | 0 | 1 | 1 |
| 14 | −85.59404 | 33.70745 | 360 | 0 | 0 | 1 | 1 |
| 15 | −85.62832 | 33.67281 | 427 | 0 | 1 | 1 | 2 |
| 16 | −85.87318 | 33.40451 | 460 | 2 | 0 | 0 | 2 |
| 17 | −85.93159 | 33.36097 | 440 | 0 | 0 | 1 | 1 |
| 18 | −86.02572 | 33.33344 | 313 | 0 | 1 | 0 | 1 |
| 19 | −87.36352 | 34.23058 | 273 | 0 | 0 | 1 | 1 |
| 20 | −85.70074 | 33.56059 | 425 | 1 | 0 | 0 | 1 |
| 21 | −87.38140 | 34.29811 | 279 | 0 | 0 | 1 | 1 |
| 22 | −87.33273 | 34.41979 | 321 | 1 | 0 | 0 | 1 |
| 23 | −87.27680 | 34.17659 | 248 | 1 | 0 | 0 | 1 |
| 24 | −85.58357 | 34.45540 | 395 | 1 | 0 | 0 | 1 |
| 25 | −85.59611 | 34.55167 | 526 | 0 | 1 | 0 | 1 |
| 26 | −85.67106 | 34.35716 | 392 | 0 | 1 | 0 | 1 |
| 27 | −85.45730 | 33.96340 | 300 | 1 | 0 | 0 | 1 |
| 28 | −85.84679 | 34.14676 | 188 | 1 | 0 | 0 | 1 |
| 29 | −85.26428 | 34.12260 | 232 | 1 | 0 | 0 | 1 |
| 30 | −85.81731 | 33.46215 | 485 | 1 | 0 | 0 | 1 |
| 31 | −84.71650 | 34.15014 | 272 | 0 | 0 | 1 | 1 |
| 32 | −83.10755 | 34.86200 | 536 | 2 | 0 | 0 | 2 |
| 33 | −83.05563 | 35.01376 | 887 | 1 | 0 | 0 | 1 |
| 34 | −83.08929 | 34.94523 | 744 | 1 | 0 | 0 | 1 |
| 35 | −83.12841 | 34.80557 | 481 | 0 | 0 | 1 | 1 |
| 36 | −83.22783 | 34.72782 | 394 | 2 | 1 | 0 | 3 |
| 37 | −83.31242 | 34.77755 | 469 | 1 | 0 | 1 | 1 |
| 38 | −83.29258 | 33.72088 | 132 | 2 | 0 | 2 | 4 |
| 39 | −86.07201 | 33.20150 | 291 | 1 | 0 | 0 | 1 |
| 40 | −84.71137 | 34.87866 | 354 | 1 | 0 | 0 | 1 |
| 41 | −84.65486 | 34.93135 | 485 | 0 | 1 | 0 | 1 |
| 42 | −84.33880 | 34.77507 | 730 | 1 | 0 | 0 | 1 |
| 43 | −84.25093 | 34.68311 | 810 | 1 | 0 | 0 | 1 |
| 44 | −83.73265 | 34.74192 | 766 | 0 | 0 | 1 | 1 |
| 45 | −83.51849 | 35.65682 | 780 | 1 | 0 | 0 | 1 |
| 46 | −83.35717 | 35.70232 | 653 | 1 | 0 | 0 | 1 |

| 47 | −83.31077 | 35.52117 | 666 | 1 | 0 | 0 | 1 |
| --- | --- | --- | --- | --- | --- | --- | --- |
| 48 | −83.21343 | 35.77140 | 575 | 1 | 0 | 0 | 1 |
| 49 | −83.66993 | 35.61933 | 593 | 0 | 1 | 0 | 1 |
| 50 | −84.69140 | 34.75931 | 804 | 1 | 0 | 0 | 1 |
| 51 | −85.49764 | 33.91858 | 257 | 1 | 0 | 0 | 1 |
| 52 | −84.62477 | 35.10896 | 530 | 0 | 0 | 1 | 1 |
| 53 | −82.07211 | 36.31100 | 648 | 0 | 0 | 1 | 1 |
| 54 | −80.54509 | 37.34757 | 1121 | 1 | 0 | 0 | 1 |
| 55 | −78.78368 | 38.12902 | 814 | 1 | 0 | 0 | 1 |
| 56 | −78.64308 | 38.29123 | 761 | 1 | 0 | 0 | 1 |
| 57 | −78.18149 | 38.80508 | 755 | 1 | 0 | 0 | 1 |
| 58 | −78.34060 | 38.62592 | 1032 | 1 | 0 | 0 | 1 |
| 59 | −79.44785 | 38.07403 | 669 | 0 | 1 | 0 | 1 |
| 60 | −79.34980 | 38.04052 | 784 | 1 | 0 | 0 | 1 |
| 61 | −87.52677 | 35.39384 | 304 | 1 | 0 | 0 | 1 |
| 62 | −84.74478 | 36.12452 | 378 | 1 | 0 | 0 | 1 |
| 63 | −84.48829 | 36.13606 | 496 | 1 | 0 | 0 | 1 |
| 64 | −85.49971 | 34.84695 | 315 | 1 | 0 | 0 | 1 |
| 65 | −88.19299 | 34.60502 | 177 | 1 | 0 | 0 | 1 |
| 66 | −84.24760 | 35.34883 | 327 | 1 | 1 | 0 | 2 |
| 67 | −84.19383 | 35.34534 | 425 | 1 | 0 | 0 | 1 |
| 68 | −83.59187 | 35.32969 | 593 | 1 | 0 | 0 | 1 |
| 69 | −82.48742 | 35.59535 | 722 | 1 | 0 | 0 | 1 |
| 70 | −82.74536 | 36.70494 | 460 | 1 | 0 | 0 | 1 |
| 71 | −79.59341 | 37.44090 | 692 | 1 | 0 | 0 | 1 |
| 72 | −79.94446 | 37.21840 | 473 | 0 | 0 | 1 | 1 |
| 73 | −79.97707 | 37.98723 | 508 | 1 | 0 | 0 | 1 |
| 74 | −79.20190 | 38.60225 | 591 | 1 | 0 | 0 | 1 |
| 75 | −79.62846 | 38.00583 | 440 | 0 | 0 | 1 | 1 |
| 76 | −78.91022 | 38.89373 | 589 | 1 | 0 | 0 | 1 |
| 77 | −82.49056 | 38.81387 | 277 | 1 | 0 | 0 | 1 |
| 78 | −84.75872 | 36.01773 | 572 | 1 | 0 | 0 | 1 |
| 79 | −84.91430 | 36.05598 | 588 | 0 | 1 | 0 | 1 |
| 80 | −84.71430 | 36.47398 | 479 | 1 | 0 | 0 | 1 |
| 81 | −79.68214 | 37.47978 | 759 | 1 | 0 | 0 | 1 |
| 82 | −84.45749 | 36.84983 | 412 | 1 | 0 | 0 | 1 |
| 83 | −84.42480 | 36.91024 | 336 | 1 | 0 | 0 | 1 |
| 84 | −83.76402 | 36.10415 | 399 | 1 | 0 | 0 | 1 |
| 85 | −83.89043 | 36.37519 | 490 | 1 | 0 | 0 | 1 |
| 86 | −83.69725 | 36.60349 | 352 | 1 | 0 | 0 | 1 |
| 87 | −83.74413 | 36.72807 | 390 | 1 | 0 | 0 | 1 |
| 88 | −83.21425 | 36.92808 | 767 | 1 | 0 | 0 | 1 |
| 89 | −82.48692 | 36.49101 | 427 | 1 | 0 | 0 | 1 |
| 90 | −82.30345 | 37.28378 | 549 | 0 | 1 | 0 | 1 |
| 91 | −82.99386 | 37.24096 | 318 | 1 | 0 | 0 | 1 |
| 92 | −81.53171 | 36.88458 | 731 | 1 | 0 | 0 | 1 |
| 93 | −85.69242 | 33.57481 | 344 | 0 | 0 | 1 | 1 |
| 94 | −84.63633 | 33.76154 | 295 | 1 | 0 | 0 | 1 |
| 95 | −82.49127 | 35.60575 | 770 | 1 | 0 | 0 | 1 |
| 96 | −82.71758 | 35.44758 | 1205 | 1 | 0 | 0 | 1 |
| 97 | −82.58961 | 35.21877 | 809 | 1 | 0 | 0 | 1 |
| 98 | −84.24123 | 35.34314 | 413 | 1 | 0 | 0 | 1 |
| 99 | −84.11201 | 35.39665 | 553 | 1 | 0 | 0 | 1 |
| 100 | −84.33586 | 35.20793 | 513 | 1 | 0 | 0 | 1 |
| 101 | −84.45322 | 35.03918 | 614 | 0 | 0 | 1 | 1 |
| 102 | −84.60815 | 35.14822 | 588 | 1 | 0 | 0 | 1 |
| 103 | −80.06615 | 36.78941 | 386 | 1 | 0 | 0 | 1 |
| 104 | −85.12241 | 35.17524 | 231 | 0 | 0 | 1 | 1 |

| 105 | −83.74414 | 34.38905 | 356 | 0 | 0 | 1 | 1 |
| --- | --- | --- | --- | --- | --- | --- | --- |
| 106 | −82.84724 | 35.85284 | 656 | 1 | 0 | 0 | 1 |
| 107 | −82.84973 | 36.08371 | 408 | 1 | 0 | 0 | 1 |
| 108 | −82.44664 | 36.10384 | 522 | 1 | 0 | 0 | 1 |
| 109 | −81.27322 | 36.12973 | 373 | 0 | 1 | 0 | 1 |
| 110 | −80.26266 | 36.39726 | 511 | 0 | 0 | 1 | 1 |
| 111 | −81.06851 | 36.37937 | 412 | 0 | 0 | 1 | 1 |
| 112 | −81.29543 | 35.20810 | 271 | 0 | 0 | 1 | 1 |
| 113 | −81.85325 | 34.87066 | 191 | 0 | 0 | 1 | 1 |
| 114 | −83.04584 | 34.49625 | 200 | 0 | 0 | 1 | 1 |
| 115 | −83.67732 | 37.77913 | 256 | 1 | 0 | 0 | 1 |
| 116 | −83.09057 | 37.75175 | 292 | 0 | 0 | 1 | 1 |
| 117 | −82.72829 | 37.71582 | 213 | 1 | 0 | 0 | 1 |
| 118 | −82.82529 | 38.05997 | 209 | 1 | 0 | 0 | 1 |
| 119 | −82.42619 | 38.30313 | 186 | 1 | 0 | 0 | 1 |
| 120 | −79.95090 | 36.43191 | 256 | 1 | 0 | 0 | 1 |
| 121 | −82.38316 | 38.02512 | 402 | 1 | 0 | 0 | 1 |
| 122 | −82.01469 | 37.88885 | 260 | 1 | 0 | 0 | 1 |
| 123 | −81.84275 | 38.18754 | 201 | 1 | 0 | 0 | 1 |
| 124 | −81.66953 | 38.26121 | 267 | 1 | 0 | 0 | 1 |
| 125 | −81.57557 | 38.65200 | 241 | 1 | 0 | 0 | 1 |
| 126 | −81.34470 | 38.77533 | 240 | 1 | 0 | 0 | 1 |
| 127 | −80.65887 | 38.63269 | 394 | 1 | 0 | 0 | 1 |
| 128 | −80.53176 | 38.82579 | 284 | 0 | 1 | 0 | 1 |
| 129 | −80.83122 | 37.50894 | 550 | 1 | 0 | 0 | 1 |
| 130 | −79.31298 | 36.78146 | 228 | 0 | 1 | 0 | 1 |
| 131 | −79.52662 | 37.07664 | 309 | 0 | 0 | 1 | 1 |
| 132 | −79.60274 | 37.09430 | 282 | 0 | 1 | 0 | 1 |
